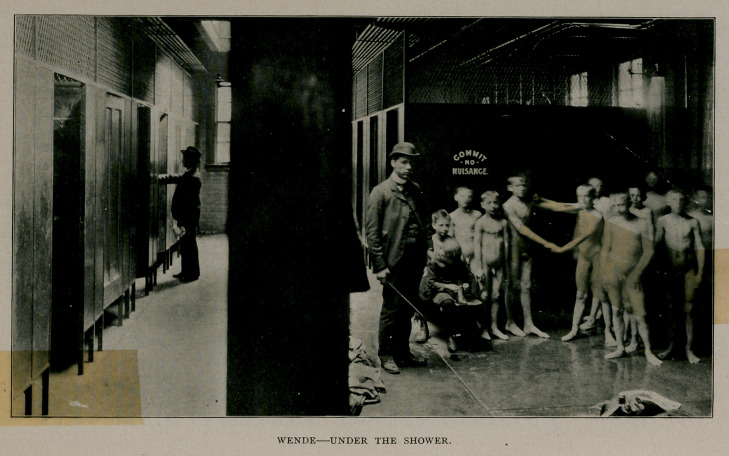# Establishment of the First Free Municipal Bath House at Buffalo, N. Y.1Read before the Medical Society of the County of Cayuga, at Auburn, N. Y., May 10, 1900.

**Published:** 1900-06

**Authors:** Ernest Wende

**Affiliations:** Health Commissioner, Buffalo, N. Y.


### ESTABLISHMENT OF THE FIRST FREE MUNICIPAL BATH HOUSE AT BUFFALO, N. Y.[1]

By ERNEST WENDE, M. D.,
Health Commissioner, Buffalo, N. Y.

IF THE prophecy of free public baths be fulfilled, its accomplishment will be largely due to the persistent efforts, and enlightened work of that eminent physician, Dr. Simon Baruch, the pioneer of scientific hydrotherapy in America. The object principally contemplated is the care of the morals, health and education of the masses, the ennobling among the poor of domestic and national life, to exchange unwholesomeness for healthfulness, and to rescue the weak, the needy and the necessitous from preventible disease. If prevention is better than cure, then the establishment of a free public bath is a greater blessing to mankind than the erection of a hospital. That prevention is better than cure is universally admitted, but the former process is too often neglected, while the latter is frequently unattainable.

It was in August, 1889, editorially in the Philadelphia *Times and Register*, that Dr. Baruch reviewed the subject of public baths, and briefly described those adopted in the German army and large German cities. Their arrangement is simple, economical, substantial, comfortable. The individual soaps himself, and is rinsed off clean by the fall of the spray-douche, the water flowing off through a latticed floor to the drain. Time, water, attendance is minimised, cleanliness obtained, danger of infection precluded by the completeness and simplicity of the system. He also advocates the rain-bath of warm water and soap as an adjunct in our public schools for the children, and also their use by the poorer class during the winter when their cleansing possibilities are limited. Such bath construction, he says, would popularize bathing, and protect against disease with but small expenditure.

A month later, at the Social Science Meeting held in Saratoga, N.Y., he read an article on Status of Water in Modern Medicine, a masterly paper, planning a bath with a capacity of 8,000 per day.

---

1. Read before the Medical Society of the County of Cayuga, at Auburn, N. Y., May 10, 1900.

Space prevents but mere allusion to his further endeavor at the New York Academy of Medicine. In the metropolis, as well, he has been the educator, elucidated and urged his proposition with the first reward of seeing it adopted in the New York Juvenile Asylum, where he is the attending physician, and, later, on a large scale, by the Association for Improving the Conditions of the Poor.

While the luxury and benefit of public baths have reached their highest stage of perfection in Germany, England, Austria and France,

SIMON BARUCH, M. D.

it remained for Buffalo, an American City, in competing for supremacy in the realisation of all the conditions desired by a cultured public, to establish a bath where the indigent, the fatigued and the unclean could find shelter and care "without money and without price."

This free public bath movement, inaugurated by Dr. Baruch, who was habitually bent on the contemplation and expectation of good municipal housekeeping, would have received earlier recognition, its utility extended, and its practicability demonstrated, had it not been blocked on several occasions by the legislature, whose primary function is often not for the good of the city, but to gain some private ends, from interested motives, as the multiplication of offices, the fixing of compensation, or the replacing of an honest and competent official by a politician or an unqualified person.

The experience of Continental Europe and Great Britain, the dictates of common sense, the vital consequence of true progress, the necessity in the cause of decency, public health and cleanliness, and a knowledge of actual conditions in Buffalo long ago emphasized the humiliating fact of the shameful need and pressing requirements of public conveniences in this country.

In the winter of 1894-5, a sub-committee of the Buffalo Charity Organisation Society, which, through personal investigation of the tenement house districts, became familiar with the fundamental principles which underlie the need of poor families that lack home conveniences, and of the working classes that require bathing facilities as a common decency of life, took under consideration the question of establishing a free municipal bath house. This committee was composed of Dr. John H. Pryor, William A. Douglas, and William Lansing, assisted by the Health Commissioner. Its members, on more than one occasion, aided and sustained those entrusted with the administration of the laws affecting public health, and through the united efforts of this committee and the Department of Health, the proposition was brought before the Common Council, and advanced to a stage where the establishment of a free public bath, by our municipality, was an assured fact, and just as the provisions became favorable, and the necessary point attained, the State Legislature, through the able assistance of Mr. Goodwin Brown, and his capable coöperator, Assemblyman George W. Hamilton, enacted the subjoined measure entitled, "An Act to Establish Free Public Baths in Cities, Villages and Towns":

Chapter 351, Laws of New York State, 1895. Section 1. "All cities of the first and second class shall establish and maintain such number of public baths as the local board of health may determine to be necessary; each bath shall be kept open not less than fourteen hours for each day, and both hot and cold water shall be provided. The erection and maintenance of river or ocean baths shall not be deemed a compliance with the requirements of this section. Any city, village or town having less than 50,000 inhabitants may establish and maintain free public baths, and any city, village or town may loan its credit or may appropriate of its funds for the purpose of establishing such free public baths.

Section 2. This act shall take effect immediately.

In framing this measure, the constitution of the State of New York recognises cities of the first class as having a population of 250,000 or over; cities of the second class, 50,000 or more, but less than 250,000; and cities of the third class less than 50,000.

Its passage was of more than municipal importance, and, as might be expected on the 19th of April, 1895, Mr. Goodwin Brown wrote to Dr. Baruch the following congratulatory letter:

It affords me pleasure to inform you that the Governor, yesterday, April 18th, signed the bill to establish and maintain free public baths

HON. GOODWIN BROWN.

in all cities of the first and second classes; cities of 50,000 inhabitants and over. I expected that it would meet with opposition in the Governor's office, on the ground that it was mandatory legislation. I am delighted to say, however, that the Governor and his advisors showed themselves exceedingly broad and liberal-minded, and the Governor did not need any urging to promptly approve the measure.

"It affords me pleasure to say that the matter which you gave me enabled me to lay before the committee unanswerable arguments, and to you I largely ascribe the credit of the most important measure of the session."

Thus, by reason of the voluntary efforts of Baruch, Brown and Hamilton, a really comprehensive measure was placed on the statute book, and through the steady perseverance of Pryor, Douglas and Lansing, and the local health official, who recognised the fact that every community has an interest in the health and strength of its individual members, and that concerted action is necessary in order to obtain practical results, the City of Buffalo was prepared to take immediate action in the direction of this improvement and much needed reform.

Now placed upon the shrine of progressive municipalism, money was appropriated, plans were ordered, site selected, contracts let, and ordinances providing for attendants and management, passed, so that by January 1, 1897, the first absolutely free municipal bath-house in the world was established, completed in every detail and put into full and successful operation. It is officially known as Public Bath House No. 1, and the praiseworthiness of the public service rendered since its inauguration affords a record—173,912 baths to males, 5,824 to females, and 57,401 to children—a grand total for the three years of 236,137 baths, which would seem to justify the city's outlay.

Its patrons, recognising not merely the propriety but the necessity of bathing, know, without reference to figures, that its capacity has been overtaxed, demonstrating a much needed and entirely inadequate public convenience to exhibit the least equivocal successes and the most splendid triumphs of sanitation.

It is not only free, but open on every day in the year, from 7 a.m. to 9 p.m., excepting on Sundays and holidays, when those requiring a bath must observe the hours from 7 to 10 a.m. There is absolutely no charge for its use, no restrictive classification of users, soap and towels are gratuitous, with an unlimited supply of water, both hot and cold, the only restriction imposed being, necessarily, one of time—namely, that bathers cannot occupy an apartment longer than twenty minutes. Additionally, facilities are offered to wash and dry underclothing, so that the equipment of the establishment sends forth those which patronise it cleansed both in person and clothing.

This is at variance with the customs that prevail at the European municipal bath houses, where, in every instance, for equal bathing facilities, a fee is demanded. This is the case in Glasgow, Manchester, London, Birmingham; in fact, in all British towns where public baths have been erected. It is, likewise, true of Paris, Berlin, Vienna, Munich, Hanover, Göttingen, Frankfort, and in all other cities of France, Germany and Austria.

To illustrate, in Glasgow, where the public baths have cost more than $600,000 and the bathers exceed 450,000 a year, a charge of two-pence is made for the use of the swimming bath, and a trifle more for private baths, with special rates for school children. In Frankfort, for instance, the price per bath is 2½ cents, including towel and soap, but the quantity of water allowed is measured and limited to 42 quarts.

It seems that in our American cities, too, it has been the

BUFFALO FREE PUBLIC BATH HOUSE NO. 1.

accredited rule to charge for these necessary innovations that improve the health and social condition of the people. For example, at the public baths in New York City, a fee of 5 cents is asked for their use. However, "tickets are granted on the most favorable terms to institutions, societies, churches and donors to the association's fund, so that the advantages of the bath may be wide-spread and extended."

Therefore, to Buffalo belongs the distinction of being the first municipality to grant to the poor, the needy and the working classes, unconditional and without charge, bathing facilities *pro bono publico*.

It is hardly necessary to say that in the selection of the site, density of population, overcrowded tenements of the poor, and cheap lodging houses, where the transient and shifting find accommodation, were regarded as the important desiderata. It is located in Police Precinct No. 1, which has an area of only 0.86 square mile or about 2 per cent. of the entire area of Buffalo, yet, according to the police census of 1895, it has a population of 20,587 or 6 per cent. of the entire population, and this notwithstanding the fact that a very large portion of the precinct is taken up by railroad tracks, the Terrace, canal, docks, depots, public buildings, stores and manufacturing establishments.

Not long since, an investigation of tenements in this section was begun by Miss Marion I. Moore, assistant secretary, Charity Organisation Society, who found that 2,784 persons were living in 72 tenement blocks, of whom 1,198 were parents; 1,263 children and 323 boarders. 128 families were living in one room per family; 203 in two rooms, and 135 in three rooms. As bathing facilities, and, indeed, privacy of any kind, do not exist among these tenants, the establishment among them of a free public bath house is a real necessity. The wisdom of the locality selected was shown from the experiences of the lack of conveniences and facilities required, in that the waiting room was constantly crowded, in spite of the existence of fourteen compartments, into and out of which the bathers were hustled every twenty minutes. With this management, the capacity of the fourteen baths is capable of accommodating forty-two persons an hour. Besides, there is a large open space for children, provided with six showers, and in which as many as twenty boys have been domiciled at one time.

The lot upon which the bath house is located is triangular in shape, having a frontage on the Terrace of fifty feet. The building is of brick exterior, as shown in photograph No. 1, and was designed by Lansing & Beierl. It is two stories high in front, with living apartments for attendants in the upper one, and one story in rear with basement under all. A reference to the ground plan will show that the interior arrangement of the building consists of a waiting room, wash-room, toilet-room, infants' bath-room, and fourteen distinct bathing apartments, all on the first floor, while, on the second are found the quarters of the attendants, possessing advantages on the score of economy, safety, privacy and convenience.

The wash-room contains three laundry tubs of concrete, and a patent drying closet which, on economical grounds, affords an

GROUND PLAN OF BUFFALO FREE PUBLIC BATH HOUSE NO. 1.

opportunity of overcoming difficulties when the bather has but one suit of undergarments which have become soiled—a God-send to one possessing such a meagre wardrobe. The infants' bath-room has a porcelain bath-tub and is for children too young to go under the showers. One must rejoice to think that the little child of the tenament house district may enjoy such a luxury. In the bathing apartments, the walls are of brick, coated with enamel paint, the floors of concrete, and the partitions of slate in iron frames, all out of consideration that sanitation by water may go wrong in proportion to the defects in construction of absorbent or nonabsorbent material.

The system adopted is that of the rain-bath and inclined shower delivering water at a maximum temperature of 100 degrees, under the control of the bather as regards volume and decrease of temperature. This has fourteen individual alcoves, and six showers in one large open space. Each alcove is divided into two parts, the dressing room and bathing room, separated by slate partitions. The shower room has a depression in the floor, of six inches deep; all angles being rounded off and supplied with waste perforation, of adequate capacity to carry off the water as fast as delivered by the shower; thus water rises about four inches making a foot bath. The bather stands erect in the bath and the shower strikes the body only from the head down. The sanitary arrangement of the antiquated tub baths sinks into insignificance, when compared with this regulation or system.

The building for wholesomeness and comfort is heated by steam, with ample provisions for its employment as a cleansing and disinfecting agent, the practical advantage of which, in an institution of this kind, cannot be over-rated, and, as a matter of safety in the bathing-rooms, the coils are suspended from the ceiling, out of reach of the bathers. Furthermore, means for flushing with water are also provided.

Experience has shown that these rain-baths concern public hygiene, that their use is a necessity to be regarded as a religious observance, symbolic of practical christianity. The advantages which affect the animal economy, the home and the public, by virtue of their purifying and exhilarating sprays are:

1st Educational.—The teaching of personal cleanliness, morality and self-respect among the poorer classes, where bathing is neglected from lack of means; many being susceptible to the salutary impressions produced, desire and soon adopt like conveniences at home.

WENDE—WAITING ROOM AND OFFICE, PUBLIC BATH NO. 1.

2d Sanitary.—Owing to the structure and mechanism, it is always scrupulously clean; no water to defile; no filthy tubs to scour; no dirty floors to scrub; no possibility of contagion.

3d Economical.—Attributable to its appliances and fittings, less space is required, less water is needed, less time for bathing necessary; the cost of tubs avoided; the expense for their maintenance saved; the detention, for tubs to fill and empty, spared.

4th Invigorating.—From the tonic and refreshing action produced by the shower, causing a concussion of the water against the skin, having the effect of a powerful massage, very much unlike the depression that enfeebles after a warm tub-bath.

5th Thorough.—From the modus operandi, the process of applying water to the erect body by means of the douche or shower, the best desired effects of cleanliness are obtained.

The consequence is, that amid the universal striving for improvement, sanitation is perpetually changing. Comparatively insignificant as this advance may seem, when measured by the period of the labors, and the number of the laborers, it is something to have to record a positive gain, however small, in a field of so much importance that is so often neglected. Respecting the management of the bath-house which is subjected to continuous care and daily observation of a keeper and his wife, and one assistant, under the supervision and approval of the Department of Health, the public is allowed to bathe with as little technicality as possible.

The following are the hours regulating the admission of male and female bathers: Week days—men and boys—7 a.m. to 12 m., and 5 p.m. to 9 p.m.; week days—women and girls—12m. to 5 p.m.; Sundays and holidays (for men and boys only) 7 to 10 a.m., and for their guidance as to deportment, the succeeding simple rules have been compiled in English, German, Polish and Italian and conspicuously posted :

RULES.

1. Smoking prohibited.
2. Swearing or obscene language not allowed.
3. No intoxicated person allowed in the building.
4. Walls, furniture or property must not be defaced or injured.
5. Soiled clothing must be taken away by the bather.
6. Towels must be returned to the keeper or matron.
7. Unused soap must be deposited in a receptacle provided for that purpose.

WENDE—UNDER THE SHOWER.

8. No bather permitted to occupy a compartment more than twenty minutes.

Statistics pertaining to Free Public Bath House No. 1, Buffalo, N.Y., are as follows:

| | |
|---|---|
| Cost of site | $6,500.00 |
| " " building | 8,000.00 |
| " " equipment | 300.00 |
| | $14,800.00 |

| Cost of Maintenance. | 1897. | 1898. | 1899. |
|---|---|---|---|
| Salary of keeper | 500.00 | 500.00 | 500.00 |
| " " matron | 400.00 | 400.co | 400.00 |
| " " assistant keeper | 200.00[1] | 480.00 | 480.00 |
| Cost of soap | 232.77 | 141.56 | 102.74 |
| " " coal | 275.35 | 330.30 | 300.00 |
| " " furnishing and laundering towels | 384.37 | 400.02 | 406 18 |
| " " incidentals | . . . | 48.05 | 182.00 |
| Totals | 1,992.49 | 2,299.93 | 2,370.92 |

TABLE OF BATHS TAKEN.

| Month. | 1897. | 1898. | 1899. |
|---|---|---|---|
| January | 3,851 | 5,918 | 4,045 |
| February | 4,357 | 4,219 | 3,919 |
| March | 6,612 | 6,633 | 6,389 |
| April | 6,984 | 6,356 | 8,094 |
| May | 7,206 | 7,406 | 8,057 |
| June | 9,291 | 9,860 | 9,627 |
| July | 9,536 | 10,221 | 1c,398 |
| August | 8,773 | 9,319 | 10,829 |
| September | 7,064 | 7,372 | 5,661 |
| October | 5,720 | 5,138 | 6,172 |
| November | 3,576 | 3,261 | 4,576 |
| December | 3,903 | 3,678 | 4,026 |
| Totals | 76,873 | 78,481 | 81,793 |

Of the above baths, were taken as follows:

| | Men. | Women. | Children. |
|---|---|---|---|
| 1897 | 55,051 | 1,395 | 20,427 |
| 1898 | 58,832 | 1,841 | 17,808 |
| 1899 | 60,029 | 2,588 | 19,176 |

The wash-room and dryer was used by 1,542 persons in 1897; 2,821 persons in 1898, and 3,166 persons in 1899.

1. Five months.

It will be found, in computing the interest on the original investment, added to the amount required for maintenance, that the cost to the city has been a fraction over 2 cents per capita for bath and laundry.

I would say, finally, that the avowed policy of many municipalities is to practise a maximum of extravagance, in the belief that such extravagance benefits the poor; on the contrary, it must be borne in mind that the establishment of free public baths should bear a certain proportion to the composition, character and density of population, and that the construction of palatial buildings for this purpose, costing a $100,000 or more, thereby saddling the taxpayer with an unnecessary expense, is indefensible. Architectural pretentions lead to a deterioration in the public health and morals. To facilitate their usefulness, their exterior should be plain and modest, so as not to repel the poor and lowly, and their interior neat and clean, with no harrassing rules and regulations. Buffalo's Public Bath House No. 1 is such a structure, unpretentious and relatively inexpensive.

Again, our municipality, under the authority of the state which establishes and places, under the control of the Department of Health, free public baths, calculated to improve the social conditions of the masses, a second establishment is now in the course of erection, and by the 1st of July its completion is contemplated for indiscriminate use. It is practically modeled upon the same lines as the first, for ease, comfort and economy of administration, likewise situated in a densely crowded section of the city, only with a larger capacity for daily use by men, women and children, without alternating hours, which will meet the indications more fully in an equally beneficial and worthy manner.

471 Delaware Avenue.

## Figures and Tables

**Figure f1:**
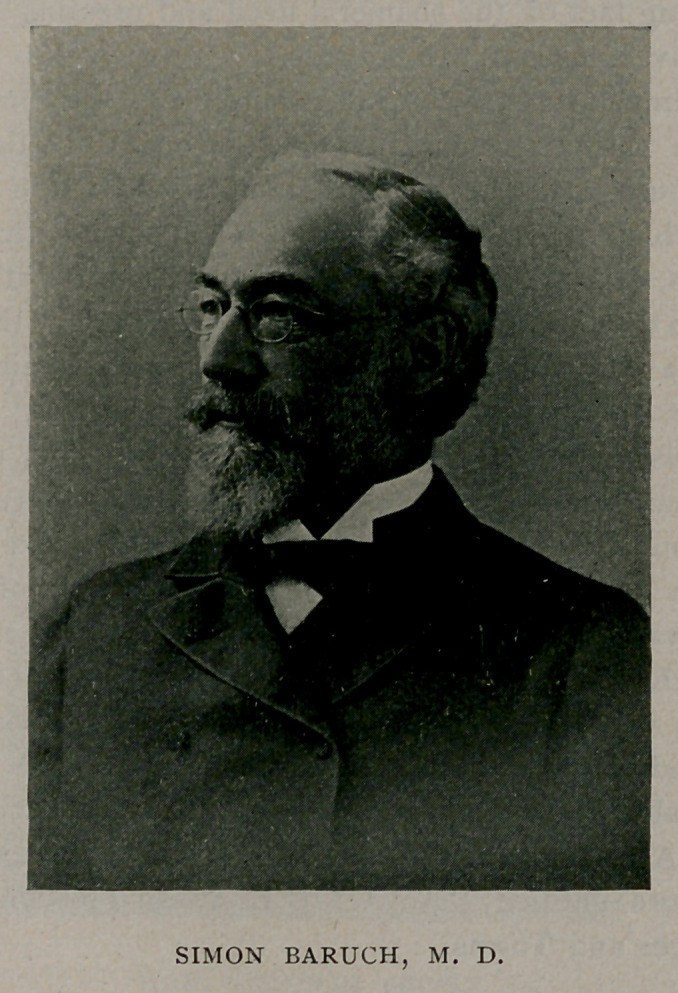


**Figure f2:**
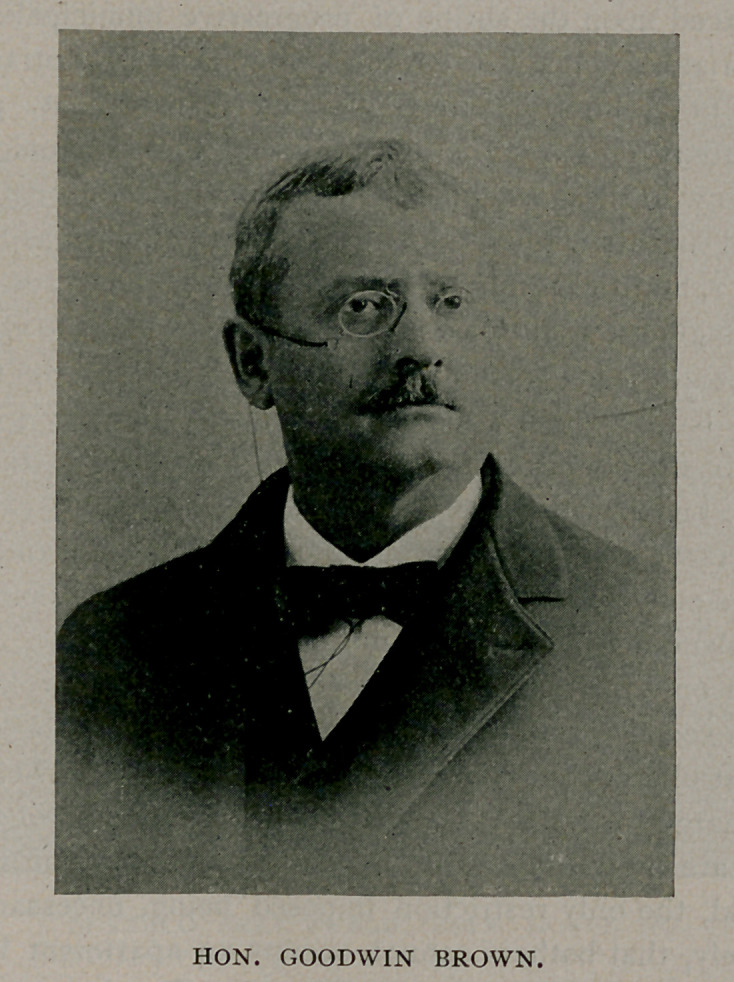


**Figure f3:**
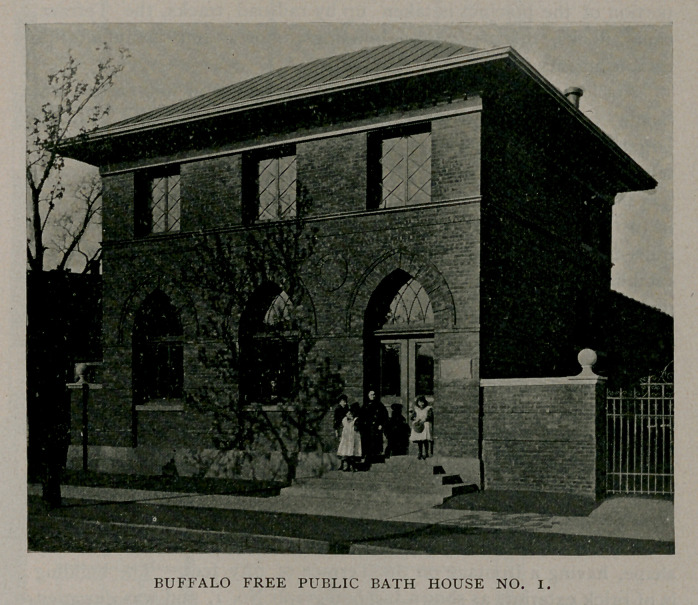


**Figure f4:**
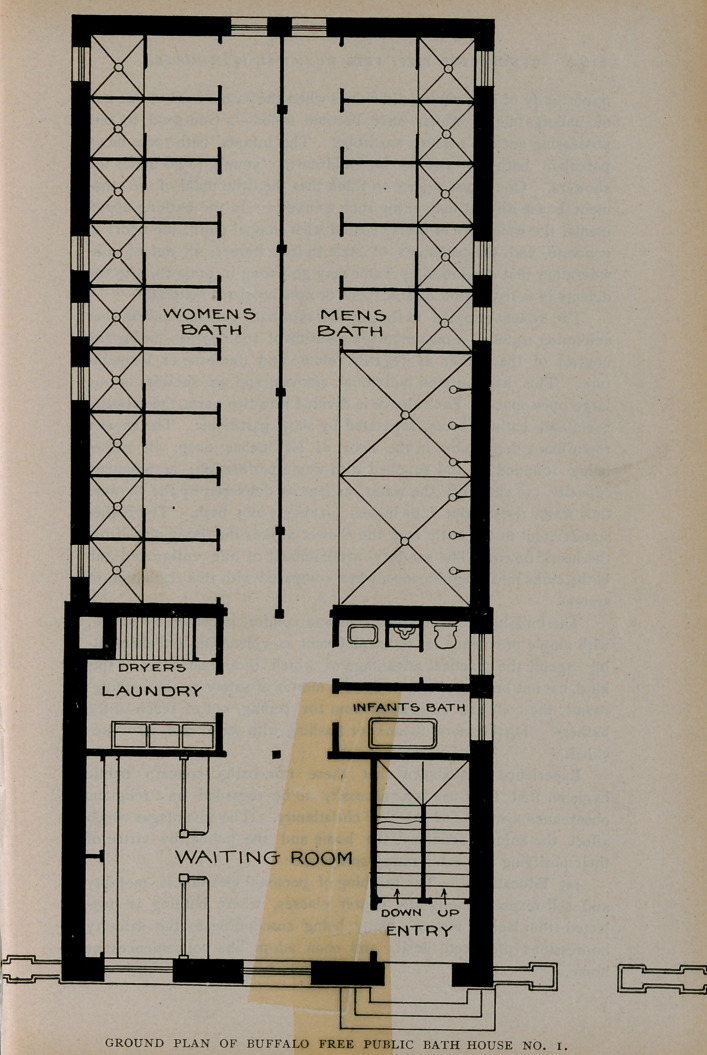


**Figure f5:**
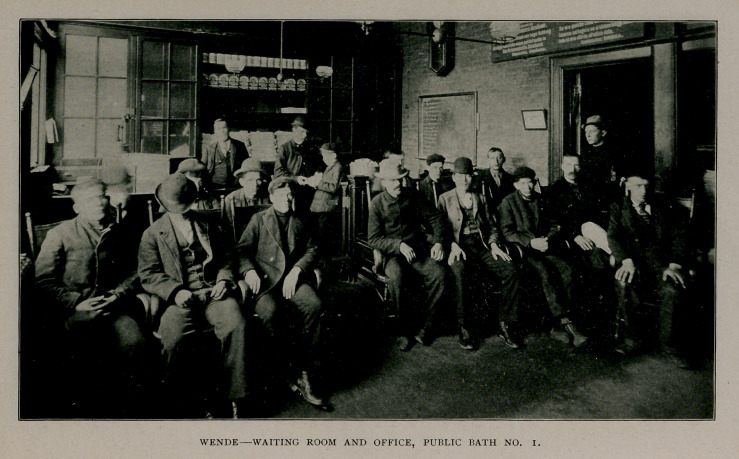


**Figure f6:**